# Polish Translation and Linguistic Validation of the SCAR-Q

**DOI:** 10.7759/cureus.52848

**Published:** 2024-01-24

**Authors:** Piotr E Janik, Jakub Opyrchał, Marcin Ambroziak, Bartłomiej Noszczyk, Marek A Paul

**Affiliations:** 1 Plastic Surgery, Department of Plastic and Reconstructive Surgery, Centre of Postgraduate Medical Education, Prof. W. Orlowski Memorial Hospital, Warsaw, POL; 2 Reconstructive Surgery, Maria Sklodowska-Curie Memorial Cancer Center and Institute of Oncology, Gliwice, POL; 3 Dermatology, Klinika Ambroziak, Warsaw, POL; 4 Plastic and Reconstructive Surgery, Doc Paul Klinika, Bytom, POL

**Keywords:** linguistic validation, cultural adaptation, translation, patient-reported outcome, scar, scar-q

## Abstract

Introduction

Patient-reported outcome measurements (PROMs) are gaining considerable popularity as tools to assess the effectiveness of the treatment in plastic surgery, being a complement to surgical outcomes. The SCAR-Q questionnaire has been recently developed for patients with surgical, traumatic, and burn scars.

Aim

The study aims to describe the process of translation and linguistic validation of the scar questionnaire (SCAR-Q) for use in Polish patients undergoing scar treatment.

Material and methods

An official Polish translation and language validation of the SCAR-Q were done in adherence to International Society for Pharmacoeconomics and Outcomes Research (ISPOR) guidelines. The process consisted of four steps: two independent forward translations, a back translation, a review of the back translation, and cognitive participant interviews.

Results

The field-tested version of the SCAR-Q consisted of 29 items across three scales measuring appearance concerns, symptoms, and the psychosocial impact of the scar. The forward translation was done by two independent translators and revealed specific difficulties in translation to the Polish language (4/29 items). The back translation showed no significant differences compared to the original English version. Cognitive debriefing interviews involved nine Polish patients with postraumatic scars, burn scars, and scars after skin tumor resection. Participants have not reported any major difficulties in understanding the content of the questionnaire.

Conclusions

The ISPOR provides a straightforward and thorough guideline for the PROMs translation process. The new SCAR-Q is an accessible and efficient PROM that can be implemented in Polish patients to assess the effectiveness of scar treatment.

## Introduction

In recent years, as health-related quality of life has become an inherent element of treatment assessment, it has led to considerable interest in patient-reported outcome measures (PROMs). It is particularly noticeable in plastic surgery, where the patients’ satisfaction and increased self-esteem can be considered as one of the determinants of treatment effectiveness or as a complement to the results of surgical procedures [1,2].

It is estimated that 266.2 to 359.5 million operations were performed worldwide, resulting in visible scars [3]. Considering countless other non-iatrogenic causes of scars due to trauma, burns, etc., it gives a slight overview of the scale of the patient population facing this problem. Individuals with significant scars may undergo emotional, functional, psychological, and social repercussions, with some cases even escalating to the point of suicide. This is particularly evident in childhood [4,5].

Until now, the subjective scar assessment tools considered mainly factors such as surface area, size, scar height, thickness, texture, pigmentation, and other physical properties in detail. Those questionnaires hadn’t put enough emphasis on the psychological burden related to the scar and thus did not provide insight into the emotional aspect of this affliction.

Recently, the scar questionnaire (SCAR-Q), a new PRO instrument, has been published [6]. It was created by contributors of other Q-series PROMs such as BREAST-Q BODY-Q or CLEFT-Q [7-9]. Thus far, four tools for assessing the perception of scars are known: the Patient and Observer Scar Assessment Scale (POSAS), the Bock Quality of Life Questionnaire for Patients with Keloid and Hypertrophic Scarring (Bock), Patient Scar Assessment Questionnaire (PSAQ), and the Patient-Reported Impact of Scars Measure (PRISM) or Vancouver Scar Scale (VSS), devised in 1990 by Sullivan et al. and primarily utilized for evaluating burn scars [10-16].

However, researchers pointed out the limitations of these surveys, which resulted in the development of the SCAR-Q [6-11]. It consists of 29 items across three scales adapted to use among children and adult patients with surgical, traumatic, and burn scars. The framework of the SCAR-Q is comprised of three overarching themes: the appearance of the scar, symptoms related to the scar, and psychosocial impact. Each scale is independently functioning and scores from 0 to 100, respectively, from worst to best. The conversion of results is based on one of the models from the item response theory (IRT)-the Rasch model, which is used to create measurements from categorical data [17]. The SCAR-Q can be used to evaluate scar perception from the moment of, e.g., burn injury, trauma, or surgery through the whole process of subsequent scar treatment.

The adaptation of the PROM questionnaire to another language should be done appropriately. It is extremely important to perform not only a proper translation but also a cultural adaptation and linguistic validation of the questionnaire. The International Society for Pharmacoeconomics and Outcomes Research (ISPOR) and the World Health Organization (WHO) have set strict guidelines to standardize but also facilitate the whole process [18,19]. Implementation of the scar-specific questionnaire, designed and developed specifically for this patient group, gives the possibility for adequate as well as objective evaluation and comparison of different scar treatment methods, helping to choose the most suitable option. Linguistic validation and the utilization of Patient-Reported Outcome Measures (PROMs) consistently consider cultural adaptation, recognizing that diverse cultures manifest unique linguistic nuances, communication styles, and contextual interpretations of phrases and words. Ensuring that the language used in a study is appropriate, fitting, and easily understandable to individuals from various cultural backgrounds is a crucial aspect of cultural adaptation. Additionally, maintaining the integrity of the study necessitates achieving semantic equivalence across languages. This implies that the meanings of the words and concepts being assessed should remain consistent across different cultural and linguistic groups. We were mindful of this in our study and made efforts to adjust, understanding that cultural adaptation plays a vital role in identifying and resolving potential disparities in meaning [18].

The study aimed to translate, linguistically validate, and culturally adapt the SCAR-Q questionnaire for Polish patients undergoing surgical procedures resulting in scarring. The necessity arose from the absence of a validated survey in Polish, and SCAR-Q stands out as a contemporary instrument for quality assessment of the scarring process. Given that the questionnaire had already been translated into various languages, including Arabic, Chinese, Dutch, Finnish, French, German, Japanese, Portuguese, Spanish, and Swedish, a Polish translation was deemed essential.

## Materials and methods

It was a nonclinical or nonexperimental survey study; therefore, a statement from the institutional ethics committee was not required. The procedures followed were based on the Helsinki Declaration of 1975, as revised in 2000, and the ethical standards of the responsible committee on human experimentation.

We obtained permission to perform a linguistic translation and validation process from the developers of the SCAR-Q (Klassen A. et al. 2018) [6]. The entire process is in line with “A Guide for Translation and Cultural Adaptation of the Q-Portfolio Questionnaires” received from the authors. An official Polish translation and language validation of the SCAR-Q was done in adherence to International Society for Pharmacoeconomics and Outcomes Research (ISPOR) guidelines [18].

The translation team was organized. It consisted of a project manager, two forward translators-native Polish speakers fluent in English-and one back translator-a sworn translator fluent in the target language.

The translation process consisted of four steps: 1) two independent forward translations, 2) back translation, 3) back translation review, and 4) cognitive interview with patients (Table 1).

**Table 1:** Cognitive Interview with patients. Cognitive interviews involve engaging patients in discussions about how they interpret, comprehend, and respond to the questions presented in an instrument.

	The Cognitive debriefing interview consisted of 3 stages
Stage 1	All participants were asked to explain what the instructions in the questions refer to. If they had any problem with understanding the instructions, they asked how the translation of the instructions can be improved so that they can understand them correctly.
Stage 2	Each participant was asked the following questions about each questionnaire item a) What is the item asking you about? If the participant does not understand what the item is asking, describe the item’s meaning to the participant and then ask the following: b) What was difficult to understand about the item? c) Is there a specific word that was difficult for you to understand? If so, what is the word? d) How can the translation of the item be improved so that you can understand the item’s meaning correctly?
Stage 3	The participants were asked the following questions about response options a) What do the different response options mean? b) Describe the difference between the response options. If the patient does not understand the differences between the meanings of the response options, describe the differences and then ask the following: c) What was difficult to understand about the response options? d) Is there a specific word that was difficult for you to understand? If so, what is the word? e) How can the translation of the response options be improved so that you can understand the meaning of the response options correctly?

**Figure 1:**
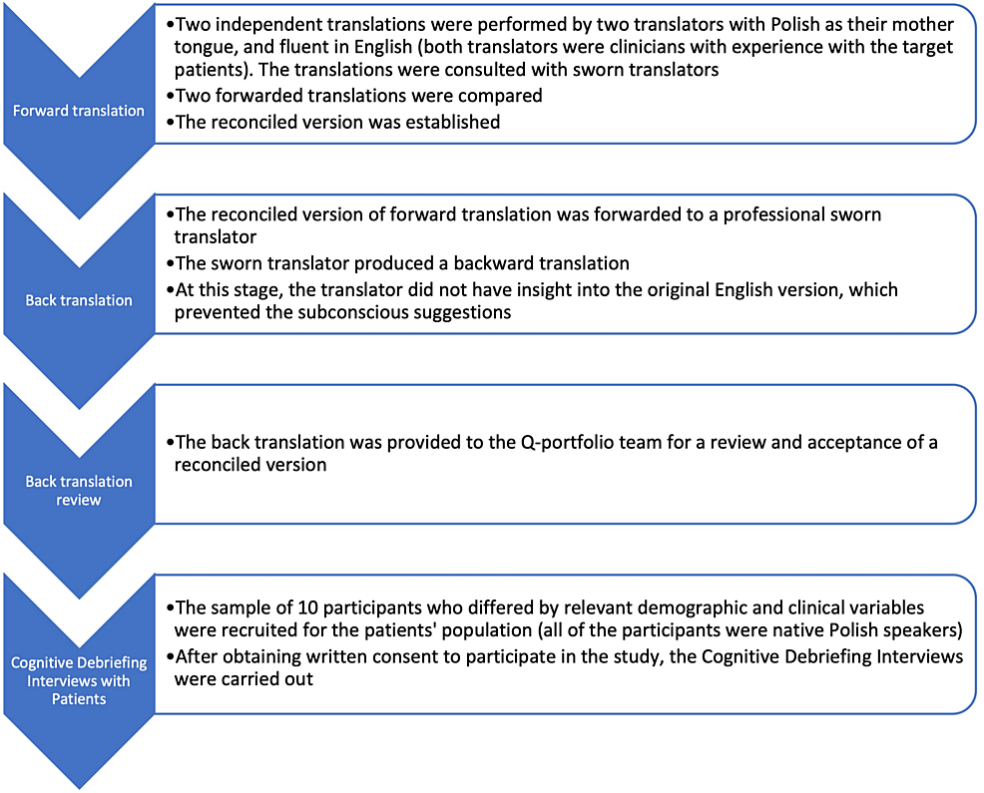
The translation process of the Scar-Q survey is delineated, with each crucial step outlined in its respective row and clarification provided where necessary.

The whole process of translation is shown in Figure 1.

After performing the cognitive debriefing interviews with patients, the draft was sent to the Q-Portfolio team for final approval by the authors. The last stage of Polish translation and language validation of the SCAR-Q was final proofreading, which led to the development of the Polish version of the questionnaire.

Statistical analysis was performed using IBM Corp. Released 2020. IBM SPSS Statistics for Windows, Version 27.0. Armonk, NY: IBM Corp. Cronbach’s alpha test was applied to measure the internal consistency of the Polish version of SCAR-Q.

## Results

Step 1: Both translators were lined up with the following scale components, such as the Instruction and Response option. They also agreed on most items. However, four translated items required discussion and arrangements. The first of them was the adjective "thick,” which means “fat” in direct Polish translation. Although the adjective “fat” is used for scars in the common language, we decided that a corresponding Polish adjective with a direct English translation as convex or bulging would be more appropriate. Another controversial adjective was "bumpy,” used in the question asking about the surface of the scar. Translator #1 translated the adjective “bumpy” using its reverse, “smooth” (“less smooth”). Translator #2 used a literal/direct translation that connotes an uneven road. After reconciling, translators decided to use “less smooth” as more suitable for referring to the flatness of the surface. Subsequently, the question about the difference between the scar and the skin color was discussed. Translator #1 proposed the noun “skin complexion,” while Translator #2 used “surrounding skin." Finally, the version with "surrounding skin" was used because the skin may vary in different areas, and it is hard to talk about complexion within hair-bearing skin (e.g., scalp).

The last discrepancy was caused by the question about the contour of the scar and its flatness. The adjective “flat” more closely matches the question about the evenness of the surface. This difference may be due to language habits; therefore, the adjective “linear” instead of “flat” (about the contour of the scar) was used.

Step 2: Back translation: This stage was performed by the sworn translator, who did not have access to the original English version of the SCAR-Q, to not affect the objective perception of the forward translation performed by our team.

Step 3: Backward translation was sent to the Q-Portfolio team for review. The authors reported no remarks. Permission to conduct the cognitive debriefing interview among patients was obtained.

Step 4: Nine participants were recruited for cognitive debriefing interviews. Table 2 presents the brief characteristics of the patients included. 

**Table 2:** Summary of the characteristics of the patients included in the study.

No	Age (Yrs)	Gender (M/F)	Diagnosis
1	25	F	Scar after lipoma resection
2	29	M	Scar after incision of furuncule
3	45	M	Scar on the chest after keloid excision with radiotherapy
4	50	M	Scar on the chest after keloid excision with radiotherapy
5	24	F	Scars after keloids (after otoplasty) excision with radiotherapy
6	41	F	Postoperative scar, arm
7	13	F	Burn scar, chest
8	33	F	Burn scar, forearm
9	25	F	Post-traumatic scar, lower limb

This stage revealed a few difficulties. First, the instruction for the question concerning feeling about the scar. In the symptom scale, it has been noticed that the instruction “HOW DOES YOUR SCAR FEEL” (original) was translated literally as "HOW IS YOUR SCAR BY TOUCH". It caused some problems with setting up the questions. It was decided to shorten the instruction to “HOW IS YOUR SCAR” which is a more suitable form of "What is your-subjective-feeling about the scar?".

On the same scale, it was also noted that the response options contain the unnecessary infinitive “to bother,” which doubled after reading the whole question. These findings were used to reduce the infinitive ‘’to bother’’ in response options. 

The last issue was the item from the symptom scale: “My scar feels like it is pulling." One of the patients understood the adjective “pulling” as “attractive” because in Polish, both words can be used alternatively to refer to the emotional sphere. Finally, the Polish form of this adjective has been changed, making it more appropriate. 

After the cognitive debriefing interviews with the patients, the Polish version of the SCAR-Q was resent with the patients’ comments and corrections for final approval. The positive response and final proofreading led to the development of the official Polish questionnaire. Our final version was linguistically validated and conceptually equivalent to the original English version, as stated by both authors as well as reviewers.

Validity and reliability are two fundamental components in the evaluation of a measurement instrument. Reliability is concerned with the ability of an instrument to measure consistently. To objectively measure this feature, we used Cronbach’s alpha, the most commonly used indicator of reliability [20]. It provides a measurement of the internal consistency of a test or scale and is scored as a number between 0 and 1. If the items are correlated to each other, the value of alpha increases. In the literature, alpha values ranging from 0.70 to 0.95 are regarded as satisfactory [21]. For our questionnaire, the calculated alpha index is 0.96, which is desirable.

## Discussion

Contemporary plastic surgery encompasses more than the procedure alone. With an increasing focus on quality of life, it becomes essential to employ linguistically validated tools for assessing patients and delivering enhanced care. Patient-reported outcome (PRO) instruments like SCAR-Q facilitate result evaluation during follow-up visits, making them integral to the overall treatment process and not to be overlooked [22].

The SCAR-Q provides a comprehensive set of scales that can now be applied to Polish patients with scars of any origin (post-trauma, burns, and post-surgical scars in adults and children). The translation process was carried out according to the strict recommendations of the International Society for Pharmacoeconomics and Outcomes Research (ISPOR) and the World Health Organization (WHO). This path is repeatedly used during the translation of PRO instruments [23-29]. Following these guidelines makes it possible to catch and correct mistakes at every stage. In our study, the Cognitive Debriefing Interview proved to us how important it is to conduct the whole process using the previously mentioned schemes. As we are aware, linguistic validation poses challenges, being time- and resource-intensive, especially under tight timelines and limited budgets. Translation quality is crucial and dependent on the skills of the translators. Engaging an adept translator is pivotal for successful outcomes. We had no major problems with the translation, but cognitive debriefing revealed a few stylistic mistakes that could lead to a complete misunderstanding of the questions. It highlights the strengths of the International Society for Pharmacoeconomics and Outcomes Research (ISPOR) guidelines.

SCAR-Q is not the first type of questionnaire used for scar assessment. One of the questionnaires concerning the scar assessment is the POSAS. It has been used for several years in clinical work [10]. It is composed of two scales: the patient scale and the observer scale. The first of them is the questionnaire, which includes seven items that measure symptoms such as itching, pain and physical and morphological features such as color, stiffness, thickness, irregularity, and overall opinion about the scar. The point of reference for the evaluation is the surrounding skin. The patient rates every item from 1 to 10. The response results are summed up, and a higher score indicates a worse outcome. The observer scale is filled out by the physician, and it includes the following items: vascularity, pigmentation, relief or texture, thickness, pliability, surface area, and overall opinion. It does not measure psychosocial factors in any way. However, it allows the patient's assessment of symptoms and the appearance of the scar from the patient's point of view.

Ziolkowski et al. performed a study that found a higher correlation between POSAS scores and the appearance and symptoms scales than between POSAS and psychosocial impact scale scores. The SCAR-Q is a rigorously developed, internationally applicable scar-specific patient-reported outcome measure that can be used to evaluate scars in research, clinical care, and quality improvement initiatives [30]. It allows not only an accurate measure of the symptoms and appearance of the scar from the point of view of patients but also the psychosocial impact of these defects.

Limitations of the study

Linguistic validation studies consistently face significant limitations. Challenges arise from cultural variations, where literal translations may miss nuances and context-specific meanings, potentially causing misinterpretation. Conceptual equivalence issues occur when certain concepts lack direct equivalents, requiring alternative expressions to convey intended meanings without bias. Response bias is another limitation, as translated surveys may yield varied responses based on cultural or linguistic disparities, introducing bias due to differences in sensitivity or relevance. Additionally, the quality of translation is crucial; inadequacies can lead to confusion, misinterpretation, and unreliable data. Meticulous attention to translation quality is vital for mitigating these limitations.

## Conclusions

The well-organized process of translation, including language validation as well as cultural adaptation, is essential to establishing a reliable PRO instrument. We translated the SCAR-Q among patients, with the diversified origin of the scar making it more relevant. The cognitive debriefing, as well as the reconciliation of both forward translations made by two independent clinicians were found useful to eliminate problems with misunderstanding the items. The proper and straightforward process of adapting the instrument to another language is of paramount importance when it comes to patient-reported outcome measurements (PROMs).
